# The Role of Non-Rubber Components on Molecular Network of Natural Rubber during Accelerated Storage

**DOI:** 10.3390/polym12122880

**Published:** 2020-11-30

**Authors:** Huifeng Zhang, Lu Zhang, Xu Chen, Yueqiong Wang, Fuchun Zhao, Mingchao Luo, Shuangquan Liao

**Affiliations:** 1 Key Laboratory of Advanced Materials of Tropical Island Resources, Ministry of Education, Hainan University, Haikou 570228, China; hfzhang69@163.com; 2 School of Life and Pharmaceutical Science, Hainan University, Haikou 570228, China; 3 School of Materials Science and Engineering, Hainan University, Haikou 570228, China; cqjsyseven@163.com (L.Z.); chen_xu1236@163.com (X.C.); yqw215@163.com (Y.W.); psfczhao@hainanu.edu.cn (F.Z.); mchluo@hainanu.edu.cn (M.L.)

**Keywords:** natural rubber, non-rubber components, network, storage hardening

## Abstract

Though the non-rubber components have long been recognized to be a vital factor affecting the network of natural rubber (NR), the authentic role of non-rubber components on the network during accelerated storage has not been fully illuminated. This work attempts to clarify the impact of non-rubber components on the network for NR during accelerated storage. A natural network model for NR was proposed based on the gel content, crosslinking density, and the non-rubber components distribution for NR before and after centrifugation. Furthermore, the effect of non-rubber components on the network was investigated during accelerated storage. The results show that terminal crosslinking induced by non-rubber components and entanglements are primary factors affecting the network formation during accelerated storage. By applying the tube model to analyze the stress-strain curves of NR, we found that the contribution of the entanglements to the network formation is larger than that of terminal crosslinking during accelerated storage. The work highlights the role of non-rubber components on the network during accelerated storage, which is essential for understanding the storage hardening mechanism of NR.

## 1. Introduction

Natural rubber (NR) from *Hevea brasiliensis* consists of 94% cis-1, 4-polyisoprene and 6% non-rubber components, such as proteins, lipids, metal ion, and so on [1,2,3,4,5]. It is well known that NR exhibits outstanding mechanical properties in comparison with synthetic cis1,4-polyisoprene rubber (IR) [6,7]. Moreover, from previous reports, it has been well-established that the prominent properties of NR can be attributed to the natural molecular network [8,9,10,11]. 

The research studies show that the non-rubber components in NR are connected with main chain through α-terminal, ω-terminal, and metal ions to form network structure [12,13,14,15]. For instance, Sakdapipanich et al. verified the existence of the non-rubber components in the natural network based on the crystallization behaviors. In addition, Guo et al. demonstrated the natural network linked by protein-based ω-terminal and phospholipids-based α-terminal [16]. Moreover, Kawaharaa et al. successfully confirmed that the outstanding mechanical properties of NR are further correlated to the non-rubber components, such as the proteins and phospholipids [17]. Despite recent progress, to the best of our knowledge, few works have been conducted for the impact of non-rubber components on the network of natural rubber during accelerated storage. Thus, unravelling how non-rubber components to regulate the network structure during accelerated storage is of wide interest, but it is challenging.

Storage hardening of NR, which is manifested by an increase in Mooney viscosity, gel content, and Wallace plasticity, has long been recognized to be a vital factor affecting the processing properties [18,19,20]. From previous results, it exposes that the storage hardening caused by the reaction between the rubber chain and so-called abnormal groups assumed to exist on main rubber chain, such as epoxide [21,22], aldehyde [23,24], and lactone [25,26]. Recently, some reports have proposed that the storage hardening is tuned by the network constructed by terminal crosslinking originating from non-rubber components [27,28,29]. However, the specific role of non-rubber components in the morphological architecture of natural rubber molecular network during storage has not been clarified. Thus, the introduction of tube model [28,30,31,32,33,34] in the current work is expected to estimate the contribution of cross-linking network and entanglement on the network polymer during storage.

Based on the issues mentioned above, the present work attempted to investigate the effect of non-rubber components on the NR network structure during accelerated storage. Initially, an authentic natural network structure model was proposed based on a comprehensive suite of crosslinking structure characterization. Furthermore, the contributions of terminal crosslinking induced by non-rubber components and entanglement on the network structure during accelerated storage were identified. The terminal crosslinking originated from the non-rubber components plays an increasingly vital role in the network structure formation during accelerated storage; thus, a mechanism for storage hardening was schematically proposed. The work holds a promising meaning in understanding the role of non-rubber components on the formation of natural network during accelerated storage.

## 2. Materials and Methods 

### 2.1. Materials

Fresh natural rubber latex was commercially supplied by China Hainan Rubber Industry Group Co., Ltd. (Haikou, China). Sodium dodecyl sulfate (SDS) and phosphorus pentoxide (P_2_O_5_) were purchased from Aladdin (Shanghai, China).

Natural rubber (NR) was prepared as following: the fresh natural rubber latex was filtered with gauze to remove impurities and preserved by adding 0.25% *v*/*v* ammonia. Subsequently, the latex was tapped into a thin film on a glass plate and drying at 30 °C for 48 h in vacuo.

Centrifuged natural rubber (CNR) was prepared through high speed centrifugation treatments. In detail, NR was dispersed in the distilled water containing 1% (*w*/*v*) sodium dodecyl sulfate (SDS) to make 25% (*w*/*w*) dry rubber content. Then, the natural rubber latex was centrifuged at 12,000 rpm/min for 60 min to separate the serum fraction. The recovered cream fraction was re-dispersed in 1% (*w*/*v*) SDS and recentrifuged at 12,000 rpm for 60 min. The resultant cream fraction was cast into thin film and dried at 30 °C. Hereafter, CNR was designated as CNR-x, where x indicates the centrifugation numbers.

The accelerated storage hardening test of NR, CNR-1, CNR-2 was conducted on the desiccator preheated at 60 °C for 30 min. Detailly, the samples and phosphorus pentoxide (P_2_O_5_) were placed in preheated desiccator and periodically extracted (0 h, 6 h, 12 h, 24 h, 36 h, 48 h). The obtained samples were denoted as NR-Y, CNR-1-Y, CNR-2-Y, where Y is the accelerated storage time.

### 2.2. Characterization

The protein content, in terms of nitrogen content, was measured by an Automatic Kieldahl Apparatus (K9860, Hanon Instruments, Jinan, China). 

The content of ester group of natural rubber was determined by the intensity ratio of carbonyl group at 1739 cm^−1^ (C=O) and unsaturated carbon absorbance at 1664 cm^−1^ (C=C) through FTIR (Spectrum One, PerkinElmer Instrument Co., Ltd., Waltham, MA, USA) [35]. 

The metal ions content data was collected by an Atomic Absorption Spectrometer (TAS-990 Super AFG, Beijing Purkinje General Instrument Co., Ltd., Beijing, China) in combination with flame method. 

The gel content was examined by dissolving accurate weight rubber in toluene at concentration of 0.1% (*w*/*v*) and then was kept in the dark for 5 days at room temperature. The insoluble fraction was separated from sol fraction by centrifugation at 14,000 rpm for 2 h and precipitated by acetone. After desiccation, the gel content was expressed as the weigh percentage of the gel fraction against the total weight.

The crosslinking density analysis was carried out by nuclear magnetic resonance (VTMR20-010V-T, Shanghai Niumag Co., Ltd., Shanghai, China) under 80 °C. 

STEM images for samples were acquired with a high-angle annular dark-field (HAADF) detector on FEI Talos F200C TEM (Thermo Fisher Scientific, Waltham, MA, USA) at an accelerating voltage of 200 kV. Ultrathin sections for STEM were prepared by a Leica EM FC7 ultramicrotome (Leica, Wetzlar, Germany) with a diamond knife at −60 °C.

The Wallace initial plasticity (P_0_) was determined by a Wallace Rapid Plastometer (MK. II, Wallace Test Equipment, Dorking, UK) according to GB/T 3510-2006.

Mooney viscosity (MS1+4) was carried out according to GB/T 1232.1-2000. The temperature of testing was 100 °C. The rubber sample was preheated at 100 °C for 1 min, followed by a shear for 4 min to measure the Mooney viscosity [36].

Stress relaxation was conducted on a DMA (Q850, TA Co., Ltd., New Castle, DE, USA) at a strain of 20% at 25 °C. The relaxation time was 25 min. Before measuring, a soak time of 3 min was applied.

Particle size and particle size distribution of gel samples were analyzed using a particle size analyzer (Zetasizer nano ZS90, Malvern Instrument, Marvin, UK). Gel of natural rubber (NR) samples were slowly dispersed in toluene.

Tensile strength measurement was performed on Gothch AI-3000 (Gotech Testing Machines Inc., Taiwan, China) at room temperature. The strain and stress were calculated from the following formulas:

(1) $$\sigma =\frac{F}{A_{0}}$$

(2) $$\varepsilon =\frac{l-l_{0}}{l_{0}}\times 100\%$$

where *σ* is the stress, and *F* denotes the observed force. *A*_0_ is related to the cross-sectional area of unstretched specimen, and *ε* is the strain, as well as l is the observed distance between the grips of extensometer on the stretched specimen. $l_{0}$ is the original distance between the extensometer.

## 3. Results

### 3.1. Non-Rubber Components and Structure of NR, CNR-1 and CNR-2

**Figure 1:**
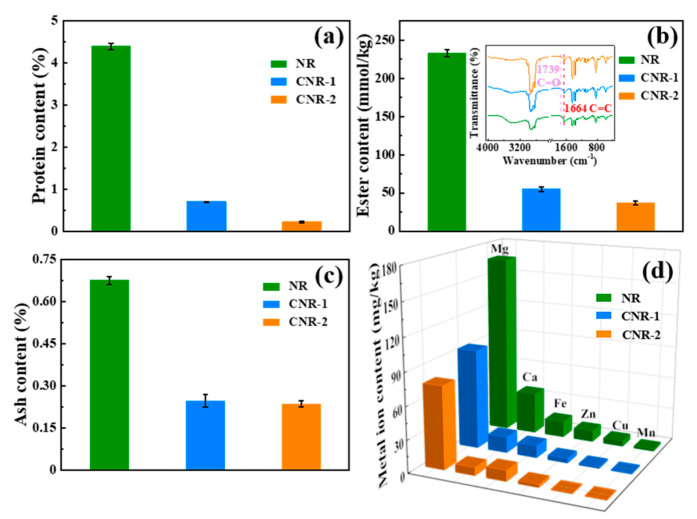
The variation of protein content (**a**), ester content (**b**), ash content (**c**), and metal ion content (**d**) in natural rubber (NR) before and after centrifugation.

The change of non-rubber components, including protein, ester, ash content, and metal ions, in NR is investigated before and after centrifugation, as shown in Figure 1. It is observed that all non-rubber components apparently decrease after treatment with centrifugation. As depicted in Figure 1a, compared with NR (4.3932%), the protein content of CNR-1 and CNR-2 is dramatically decreased to 0.7007% and 0.2237%, respectively, indicating that the protein in NR is removable through centrifugation. To accurately quantify the content of ester in NR, CNR-1, and CNR-2, the intensity ratio of carbonyl group to unsaturated carbon was assessed by FTIR (as shown in inserted Figure 1b). The content of ester in NR remarkably decreases to 54.84 mmol/kg (CNR-1) after once centrifugation, and further decreases to 36.55 mmol/kg (CNR-2) followed by double centrifugation. Ash content is measured to provide the information about the inorganic salt (phosphate or sulfate of potassium, calcium, magnesium, aluminum, copper, and other metallic elements) content of the prepared samples. From Figure 1c, the ash content is calculated to be 0.6755% (NR), 0.2473% (CNR-1), and 0.2364% (CNR-2), respectively, indicating that the inorganic salt in NR was removed by centrifugation. Furthermore, the result in Figure 1d confirms that the decrease in ash content is attributed the metal ions removal, especially Mg^2+^ and Ca^2+^. Besides, it is noted that the color of samples gradually become shallow as the centrifugation number increases (Figure S1), which, along with the above results, verifies the removal of non-rubber components in NR. The above analyses illustrate that the centrifugation treatment is a valid approach to remove the non-rubber components in NR, and the resultant content of non-rubber components in NR depends on the centrifugation number. 

**Figure 2:**
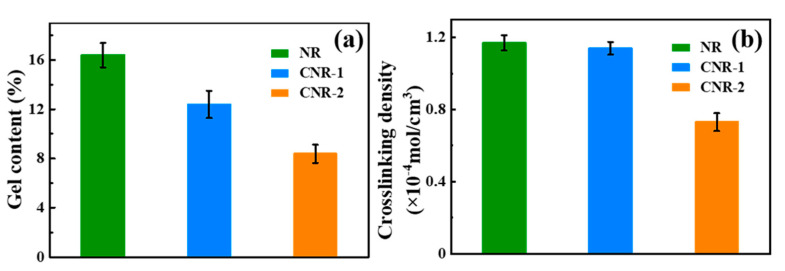
The variation of gel content (**a**) and crosslinking density (**b**) in NR before and after centrifugation.

To further investigate the impact of non-rubber components on the network of NR, the gel content and crosslinking density (Figure 2) were examined. After centrifugation, the gel content of CNR-1 (12.39%) and CNR-2 (12.39%) is obviously less than that of NR (16.39%). Simultaneously, compared with NR (1.17 × 10^−4^ mol/cm^3^), the crosslinking density of CNR-1 and CNR-2 is decreased to 1.14 × 10^−4^ mol/cm^3^ and 0.73 × 10^−4^ mol/cm^3^, respectively. The above results clearly reveal that the gel content and crosslinking density could be significantly affected by the non-rubber components, which might be due to the decomposition of crosslinking points originating from non-rubber components, such as protein, phospholipids, and metal ions. Moreover, the variation of gel content and crosslinking density is correlated to the network structure in NR. Consequently, it concludes that the non-rubber components play an important role in the construction of network structure in NR.

**Figure 3:**
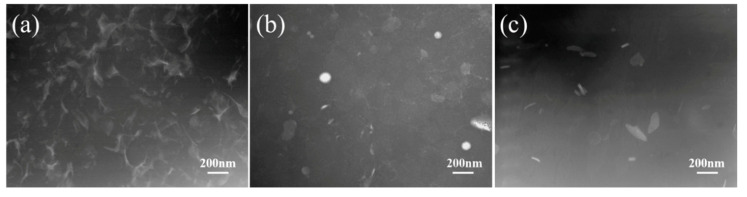
STEM images of NR (**a**), centrifuged NR (CNR)-1 (**b**), and CNR-2 (**c**).

The dispersion of non-rubber components in the rubber matrix and the variation of network are characterized by STEM (Figure 3). According to the STEM observation, phase-separated structure is found for NR and CNR, in which bright domains represent non-rubber components and gloomy domains represent molecular chain. It is clearly seen that non-rubber components distribute uniformly in the NR matrix (Figure 3a–c). Besides, the network induced by non-rubber components is observed due to intrinsic ionic bonds and H-bonding among non-rubber components [2,36,37,38,39,40,41,42]. However, the content of non-rubber components decreases significantly after centrifugation, which results in the decomposition of network (Figure 3b,c). Therefore, the existence of non-rubber components in rubber is crucial for the network formation.

**Figure 4:**
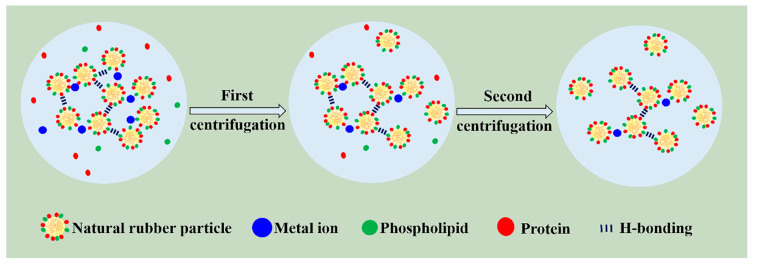
Schematic of the natural network structure in NR.

On the basis of analyses mentioned above, we tentatively proposed a novel model for natural network structure of NR, as illustrated in Figure 4. NR molecules chains comprise of long-chain isoprene units, as well postulated that α-terminal links with mono- or di-phosphate groups that associate with phospholipids, whereas the ω-terminal is a dimethylallyl group that interacts with proteins [12,43,44,45]. Meanwhile, these molecules chains could interact with each other via their terminal groups. Namely, phospholipids are linked to another phospholipid molecular in other chain ends via H-bonding or ionic bonds derived from metal ions, whereas proteins could interact with other protein molecules through H-bonding and metal ions. Moreover, proteins, phospholipids, and metal ions in NR can function as crosslinking points, inducing crosslinking network formation in terminal groups (terminal crosslinking). Notably, the hydrogen bond and ionic bond are vulnerable to breakage under centrifugation, as evidenced by the decrease of metal ion content (e.g., Ca^2^^+^ and Mg^2+^). The decrease is also responsible for the change in gel content and crosslinking density after centrifugation (Figure 2). Additionally, entanglements, which can function as crosslinking points, are a key component of natural network [39,46,47,48,49]. 

### 3.2. Crosslinking Density and Gel Content of NR, CNR-1, and CNR-2 Before and after Accelerated Storage 

**Figure 5:**
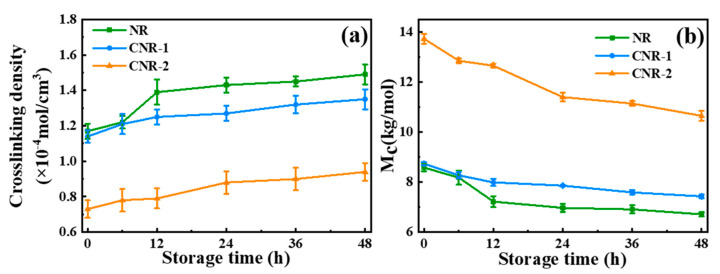
The variation of crosslinking density (**a**) and the average molecular weight between crosslinking (M_c_) (**b**) of NR, CNR-1, and CNR-2 before and after accelerated storage.

In order to assess the effect of non-rubber components on network structure of NR during accelerated storage, the accelerated storage hardening test was carried out. As reflected by the Figure S2, the variation of the Mooney viscosity and P_0_ for both NR and CNR determines the occurrence of storage hardening. Furthermore, Figure 5a,b describe the change in crosslinking density and the average molecular weight between crosslinking (M_c_) of NR, CNR during accelerated storage. An increase in crosslinking density for all samples is obtained during accelerated storage, which might be result of the molecular crosslinking. Nevertheless, after accelerated storage for 48 h, the CNR-1 (1.35 × 10^−4^ mol/cm^3^) and CNR-2 (0.94 × 10^−4^ mol/cm^3^) exhibit a lower crosslinking density compared with NR as received with the crosslinking density of 1.49 × 10^−4^ mol/cm^3^, suggesting the decrease of non-rubber components is hardly conducive to the formation of network structure during accelerated storage. The changing trend of M_c_ for NR, CNR-1, and CNR-2 in Figure 5b is in good accordance with the variation of crosslinking density, which further supports the conclusion that the non-rubber components are important in the formation of network structure during accelerated storage.

**Figure 6:**
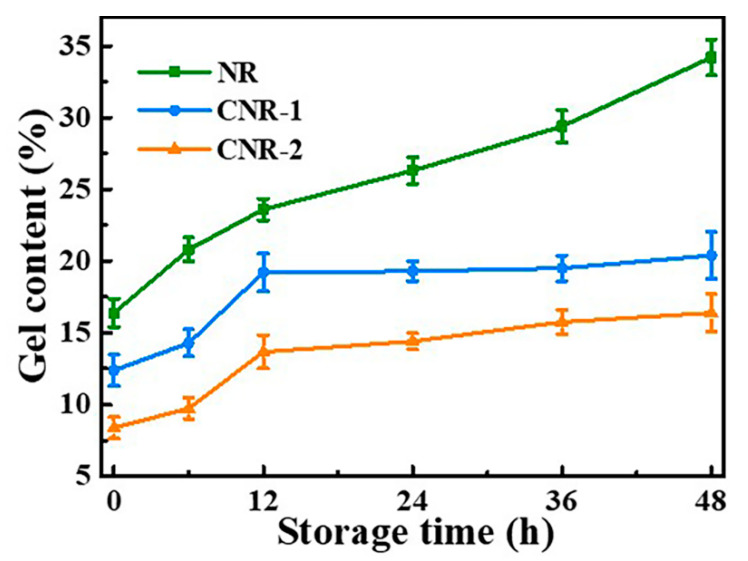
The variation of gel content of NR, CNR-1, and CNR-2 before and after accelerated storage.

Furthermore, gel content can reflect the degree of crosslinking of regional network molecules of NR samples from a macroscopic perspective [50]. The change in the gel content of NR, CNR-1, and CNR-2 during accelerated storage is evaluated and the result is displayed in Figure 6. At initial storage stage (≤12 h), the gel content of all samples gradually increases over time, which is explained by the increase in network density. When the storage time is further extended, unlike the NR, there is no significant change in gel content of CNR-1 and CNR-2. It is noted that NR has the maximum gel content and the highest crosslinking degree before and after accelerated storage, which is ascribed to that NR possesses abundant protein, ester, and metal ions (Figure 1). The results suggest that the terminals crosslinking arising from non-rubber components facilitate the gel formation.

### 3.3. Molecular Network of NR, CNR-1, and CNR-2 Before and after Accelerated Storage 

**Figure 7:**
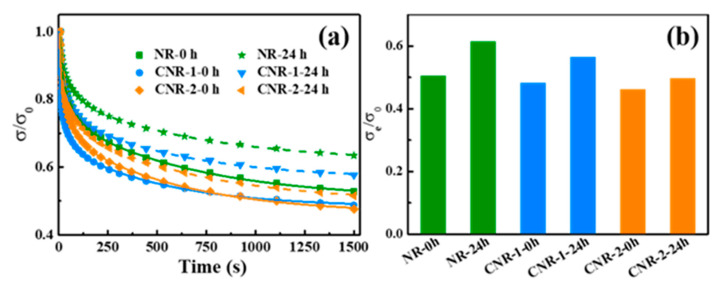
Stress relaxation curves (**a**) and residual stress (**b**) of NR, CNR-1, and CNR-2 before and after accelerated storage.

To further investigate the effect of non-rubber on the network during accelerated storage, stress relaxation, being an effective method, was analyzed. The stress relaxation curves of NR, CNR-1, and CNR-2 before and after accelerated storage are shown in Figure 7a.

The stress relaxation curve of NR-0 h tends to form a plateau after approximately 300 s, and the retention is more than 52%, which is larger than that of CNR-1-0 h (48.78%) and CNR-2-0 h (47.62%). However, the stress retention of samples obviously increases after accelerated storage. Particularly, NR-24 h has the maximum stress retention (63.00%). It can be confirmed that the more relaxation units in NR-24 h are obviously confined by the network with abundant non-rubber components. Furthermore, the Maxwell model is used to understand the stress relaxation curves [51,52,53].

(3) $$\sigma \left(t\right)=\sigma_{e}+\overset{n}{\underset{i=1}{\sum }}\sigma_{i}e^{-\frac{t}{\tau_{i}}}$$

(4) $$\frac{\sigma \left(t\right)}{\sigma_{0}}=\frac{\sigma_{e}}{\sigma_{0}}+\overset{n}{\underset{i=1}{\sum }}\frac{\sigma_{i}}{\sigma_{0}}e^{-\frac{t}{\tau_{i}}}$$

In formulas, *σ*(*t*), *σ_e_*, and *σ*_0_ are the stress in the relaxation, the equilibrium stress, and the initial stress, respectively. *σ_i_* and *τ_i_* represent the coefficient and relaxation time of the *i*th Maxwell model, respectively.

The Maxwell fit result of σ*_e/_*σ*_0_* is displayed in Figure 7b. Due to the existence of network, the movement of rubber molecular chain is limited, resulting in a greater stress and a longer time to reach equilibrium. As a result, residual stress decreases in this order: NR-24 h > CNR-1-24 h > CNR-2-24 h, NR-0 h> CNR-1-0 h > CNR-2-0 h. Moreover, compared with the *σ_e/_σ*_0_ before accelerated storage, the σ*_e/_*σ_0_ of all samples higher after accelerated storage. Notably, NR-24 h displays the largest *σ_e/_σ*_0_ and longest stress relaxation time, which is proportional to crosslinking density; therefore, NR-24 h has the largest crosslinking density, which is consistent with the results of Figure 5. 

**Figure 8:**
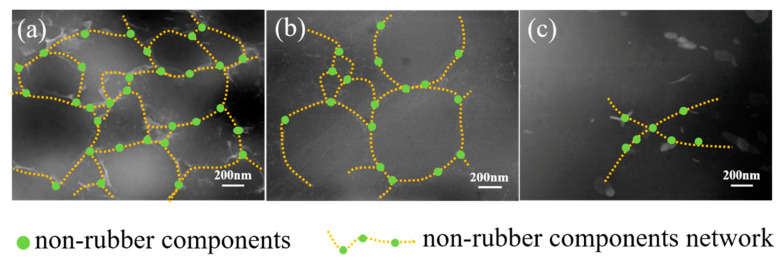
STEM images of NR, CNR-1, and CNR-2 after accelerated storage: NR-24 h (**a**), CNR-1-24 h (**b**), and CNR-2-24 h (**c**).

The network of NR, CNR-1, and CNR-2 after accelerated storage is observed by STEM, and the results are shown in Figure 8. The original STEM images without lines are shown in Figure S3. Compared with the STEM results before accelerated storage (Figure 3), it can be seen that the network density in NR increases and the network become more regular after accelerated storage, which further explain the reason for the increase in gel content and crosslinking density after accelerated storage. In addition, the particle size of NR gel is also gradually increased before and after storage (Figure S4), which is mutually verified with the STEM. However, a slight increase for network density in CNR-1 and CNR-2 is obtained after accelerated storage. 

**Figure 9:**
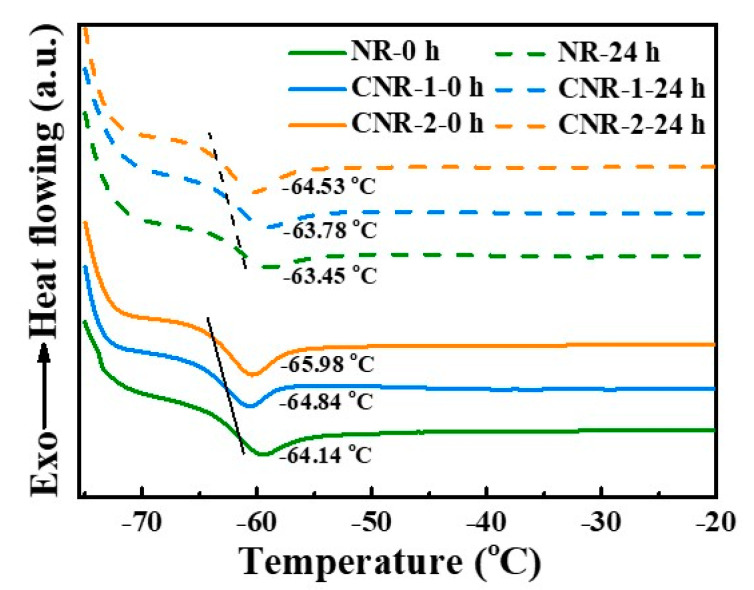
T_g_ (glass transition temperature) of NR, CNR-1, and CNR-2 before and after accelerated storage.

The difference in network further influences the glass transition temperature (T_g_) during accelerated storage. As shown in Figure 9, the T_g_ of NR, CNR-1 and CNR-2 before and after accelerated storage is measured and the results are shown in Figure 9. Compared with samples before accelerated storage, a increase in T_g_ is observed for all samples after accelerated storage, attributing that the increase in network density restricts the slip of molecular chain. Nevertheless, it is worth mentioning that the NR-24 h exhibits the highest T_g_ value (−63.45 °C) in comparison with CNR-1-24 h (−63.78 °C) and CNR-2-24 h (−64.53 °C), illustrating that the terminal crosslinking derived from non-rubber components have noticeable impact on the slip of molecular chain during accelerated storage. As a consequence, we speculate the terminal crosslinking and the state of entanglement significantly influence the slip of molecular chain, and, subsequently, the network structure during accelerated storage.

**Figure 10:**
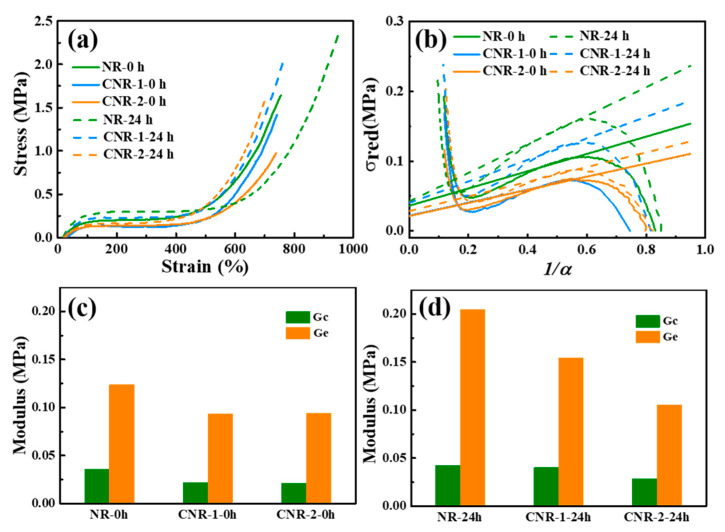
Stress-strain curves (**a**), Mooney-Rivlin plots of reduced stress (**b**) and parameters G_c_ and G_e_ (**c)**,(**d**) of NR, CNR-1 and CNR-2 before and after accelerated storage.

However, we still cannot distinguish the contribution of the terminal crosslinking and that of the entanglements to the network structure during accelerated storage. To address this problem, the stress-strain curves are first studied, they can reflect the raw NR resisting deformation and fracture when the samples are stretched. As displayed in Figure 10a, an obvious increase in stress after accelerated storage for NR, CNR-1, and CNR-2 is observed, ascribed to the increase of crosslinking density (Figure 5) during accelerated storage. Meanwhile, the NR exhibits a higher stress in comparison with CNR-1 and CNR-2 after accelerated storage. This can be explained by the fact that the existence of plentiful terminal crosslinking induced by non-rubber components and entanglements constrains the network to relax and eliminates to some extent the stress to resist deformation and fracture. The result provides another support for the above speculation that the network structure can be significantly affected by terminal crosslinking and entanglements during accelerated storage. Furthermore, the stress-strain curves are analyzed according to the tube model, which assumes an additive contribution of chemical cross-links and the entanglement [54]. Therefore, the tube model can distinguish the contribution of the terminal crosslinking and entanglements on the network by the Mooney-Rivlin (MR) law. Moreover, the tube mode is often used to describe the nonlinear elastic response of stretched polymer. Since the NR, CNR-1, and CNR-2 exhibit strongly nonlinear stress-strain relations (Figure 10a), the tube model is more suitably used to describe the elastic behaviors [27]. The reduced Mooney stress is written in the form:

σ*_red_* = σ/(α – α^−2^) = G_c_ + G_e_ × (1/α),

where σ*_red_* is the reduced stress, σ denotes the nominal stress, and α is the elongation ratio. G_c_ is the elastic modulus attributing to the contribution of crosslinks generated by the terminals; G_e_ is the modulus ascribing to the contribution of the entanglements [28,32,54]. G_c_ and G_e_ can be determined from the y-axis intercept and the slope of the fitting line, respectively.

The corresponding Mooney-Rivlin plots are shown in Figure 10b, and the fitting parameters are presented in Figure 10c,d, respectively. An obvious reduction in reduced stress (σ*_red_*) is observed at the low strain region due to the relaxation or slippage of entanglements. Following that, the reduced stress gradually increases with increasing stretching because of different strain-induced crystallization behaviors [27,54].

**Figure 11:**
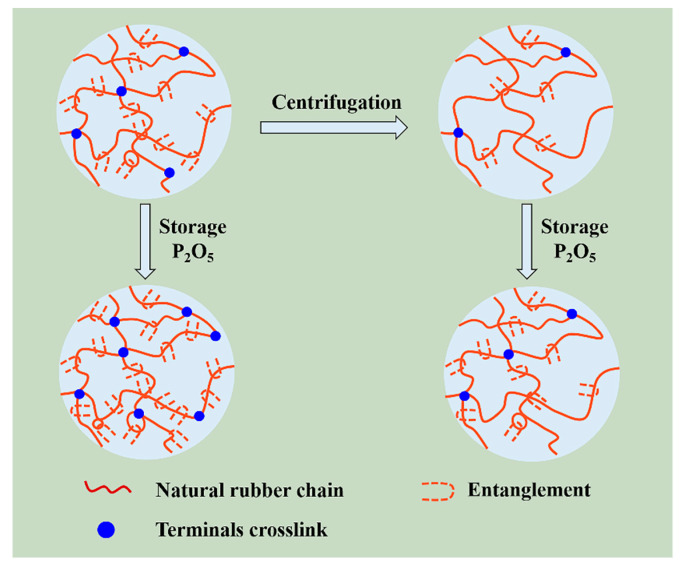
A proposed model for NR after storage hardening.

As depicted in Figure 10c,d, both G_e_ and G_c_ values are considerably decreased for CNR-1 and CNR-2, in agreement with literature reported, suggesting the G_c_ and G_e_ values depend on the content of non-rubber components. After accelerated storage, the G_e_ value increases greatly for NR, accompanied by a slightly increase in G_c_. The G_e_ and G_c_ of CNR-1 show the same trend, only lower than NR. Surprisingly, no significant change in G_c_ or G_e_ is observed for CNR-2. The different change in G_c_ and G_e_ for NR, CNR-1 and CNR-2 might be explained by the variation of non-rubber components. For NR, the presence of abundant non-rubber components is beneficial for the formation of terminal crosslinking network, especially the ionic bonds between protein and phospholipid. During accelerated storage at higher temperature, the ever-increasing entanglement caused by the NR molecular chain segments movement leads to the increase of G_e_. It is worth noting that G_e_ is much larger than G_c_ in all samples before and after accelerated storage, which indicates that the molecular chains’ entanglements play a more vital roles in contributing to the reduced stress (Figure 11), which is unlike that shown in vulcanized NR [32]. For CNR, the sharp decrease in non-rubber components is unfavorable to the formation of network constructed by the terminal crosslinking and entanglements, resulting in a slight increase for both G_c_ and G_e_ during accelerated storage. And, more notably, the resultant G_c_ and G_e_ values of CNR-1 and CNR-2 are less than that of NR after accelerated storage. Both results further testify the network structure of NR will be adjusted by the content protein, phospholipid and metal ions before and after accelerated storage.

A storage hardening mechanism based on the obtained results can be proposed (Figure 11). The non-rubber components, such as protein, phospholipid, and metal ions, affect the contributions of the terminals and the entanglements on the network structure and further impact the occurrence storage hardening. Nevertheless, although both the terminal crosslinking and entanglement are increased after accelerated storage, the entanglement dominates the formation of network during accelerated storage.

## 4. Conclusions

In this paper, we aimed to illuminate the effect of non-rubber components on the network structure during accelerated storage. Primarily, the centrifugation treatment apparently decreases the content of non-rubber components in NR, which provides a convenient and versatile method to analyses network structure in comparison with traditional enzymatic or chemical approaches. Subsequently, the natural network structure model of NR is established by the change in the protein, ester content, metal ions, gel content, and crosslinking density before and after centrifugation. Indeed, NR comprises not only long-chain cis1,4-polyisoprene but also the two end groups. The terminal groups at the rubber chain ends can be further interacted with other rubber molecular chain through non-rubber components present in NR, such as phospholipids, proteins, and metal ions, and virtually form network structure by hydrogen bonding or ionic linkage. Interestingly, we find that the hydrogen bonding or ionic linkage is easy to be breakage under centrifugation treatment, which is important for the network. Furthermore, a detailed and systemic research on the storage hardening reveal that the non-rubber components have an important influence on the network structure during accelerated storage. In addition, the tube model further testifies the contributions of terminal crosslinking induced by non-rubber components and the entanglement on the network structure. And, most importantly, entanglement dominates the formation of network during accelerated storage. To summarize, the work acquires a better understanding of the role of non-rubber components on the network structure during accelerated storage.

## Supplementary Materials

The following are available online at https://www.mdpi.com/2073-4360/12/12/2880/s1, Figure S1: Digital images of NR, CNR-1, and CNR-2; Figure S2: P_0_ and Mooney viscosity of NR, CNR-1, and CNR-2 before and after accelerated storage; Figure S3: Original STEM images of NR, CNR-1 and CNR-2 after accelerated storage: NR-24 h (a), CNR-1-24 h (b), CNR-2-24 h (c); Figure S4: Particle size distributions of gel in NR before and after accelerated storage.
