# Supplementary material for: The Role of Non-Rubber Components on Molecular Network of Natural Rubber during Accelerated Storage
Source: Polymers (Basel). 2020 Nov 30;12(12):2880. doi: 10.3390/polym12122880 (PMC7760701; doi:10.3390/polym12122880)
Supplement: Supplementary file 1 [file polymers-12-02880-s001.pdf]

polymers

Supplementary Materials

The Role of Non-rubber Components on Molecular Network of Natural Rubber During Accelerated Storage

Huifeng Zhang [1,2], Lu Zhang [3], Xu Chen [3], Yueqiong Wang [3], Fuchun Zhao [3], Mingchao Luo [3] and Shuangquan Liao [1,3]*

[1] Key Laboratory of Advanced Materials of Tropical Island Resources, Ministry of Education, Hainan University, Haikou, 570228, China;
[2] School of Life and Pharmaceutical Science, Hainan University, Haikou, 570228, China;
[3] School of Materials Science and Engineering, Hainan University, Haikou, 570228, China;
* Correspondence: lsqhnu@hainanu.edu.cn;

Received: date; Accepted: date; Published: date

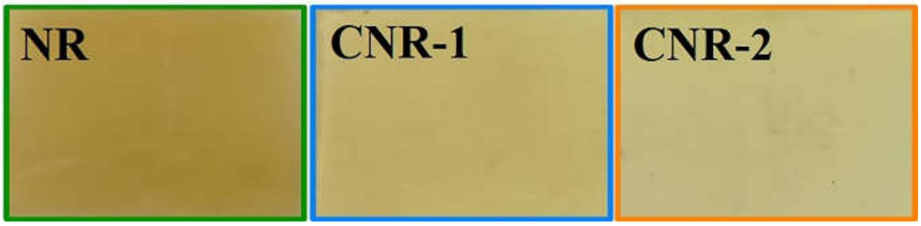

Figure S1 Digital images of NR, CNR-1 and CNR-2

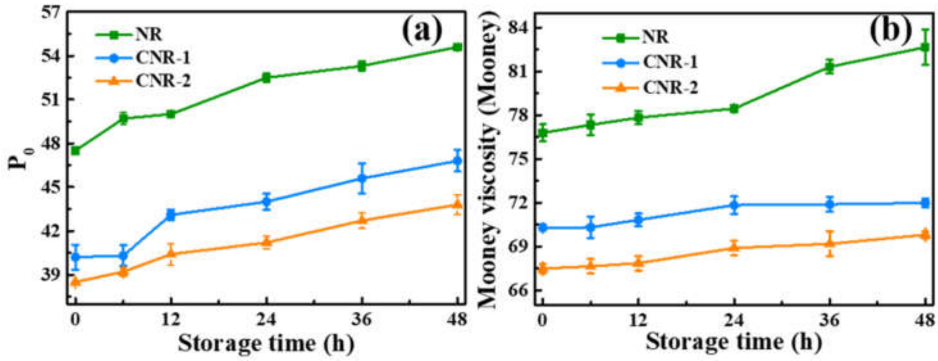

Figure S2 P_0 and Mooney viscosity of NR, CNR-1 and CNR-2 before and after accelerated storage

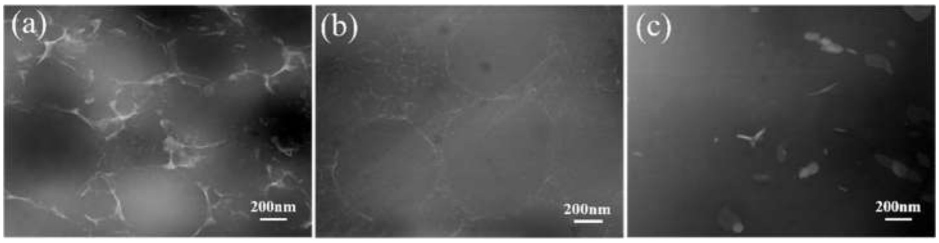

Figure S3 Original STEM images of NR, CNR-1 and CNR-2 after accelerated storage: NR-24 h (a), CNR-1-24 h (b), CNR-2-24 h (c)

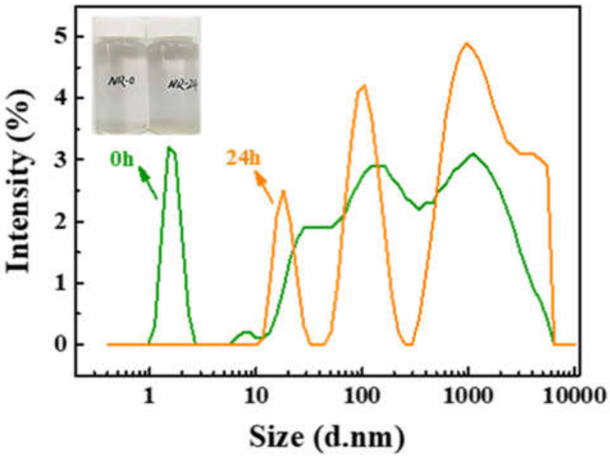

Figure S4 Particle size distributions of gel in NR before and after accelerated storage
